# Supplementary material for: Causes and consequences of fever in Amazonian pregnant women: A large retrospective study from French Guiana
Source: PLoS Negl Trop Dis. 2023 Oct 24;17(10):e0011721. doi: 10.1371/journal.pntd.0011721 (PMC10624260; doi:10.1371/journal.pntd.0011721)
Supplement: S1 Table — (DOCX) [file pntd.0011721.s001.docx]

**Supplementary table 1: comparative analysis of fever etiologies during pregnancy in west French Guiana and mainland France.**

| Causes of fever | Amazon region  (%) | Mainland France (35)  (%) |
| --- | --- | --- |
| Arboviruses | 30.2 | 0 |
| Urinary tract infections | 27.7 | 21 |
| COVID 19 | 13.2 | - |
| Chickenpox | 5.5 | 0 |
| Influenza | 3.3 | 21 |
| Gastrointestinal infections | 2.9 | 6 |
| Ear, nose, and throat disorders | 2.4 | 2 |
| Pulmonary infections | 2 | - |
| Leptospirosis | 1.8 | 0 |
| Malaria | 1.6 | 0 |
| Sexually transmitted infections | 1.4 | 1 |
| *Coxiella burnetii (Q fever)* | 1 | 0 |
| *Listeria Monocytogenes* | 0.2 | 0 |
| Unknown diagnosis | 8.5 | 15 |
