# Supplementary material for: Causes and consequences of fever in Amazonian pregnant women: A large retrospective study from French Guiana
Source: PLoS Negl Trop Dis. 2023 Oct 24;17(10):e0011721. doi: 10.1371/journal.pntd.0011721 (PMC10624260; doi:10.1371/journal.pntd.0011721)
Supplement: Attachment — Submitted filename: review.docx [file pntd.0011721.s002.docx]

General comments

In this manuscript, the authors aim to describe the causes and consequences of fever in pregnant women who delivered in a referral center of Western French Guiana. This is an important area of research since infections during pregnancy can potentially result in unfavorable maternal and fetal outcomes. The authors found that about 4% of pregnant women has fever during pregnancy and among women with known etiology for fever, arboviral infection was diagnosed in a significant proportion of patients. Although the findings of the study are important, several key issues regarding methodology and analysis need to be addressed for interpreting the findings of the study.

Major comments

1. The authors need to define their study population clearly while analyzing the findings. The objective of study was to study pregnant women with fever. It is mentioned that women were included after 18 weeks of gestation (line 102). However, the results include data from patients in first trimester also (lines 289, 290). It is also mentioned in the same lines that fever during labor has been excluded. This was not part of the exclusion criteria mentioned in methods, neither the rationale for this exclusion is explained. Did the authors mean fever AFTER delivery? Again, in lines 433-434, we read that intrapartum fever is NOT excluded and there is also a mention of a subgroup analysis, the data on which is missing. Thus, there is no clarity regarding the study population.
2. It is not clear whether more than one fever episodes in the same woman has been included or excluded. There were 940 pregnant women with at least 1 episode of fever. It is also mentioned there were 978 infections (line 286).
3. Line 297 reads that there were significant differences in pre-existing diabetes between women with and without fever, however this is not substantiated by results provided in Table 2.
4. How many women were hospitalized?
5. Table 2 shows the comparison between gestational characteristics of women with and without fever. Although statistically significant differences between gravida and parity is shown between them one wonders how to interpret these findings. Does it mean that younger women with lower parity are predisposed to infections? If so, what are the possible reasons for this? Is it related to differences in the healthcare seeking behaviors in primi versus multigravida women?
6. The study period is spanning 9 years. It is possible that some women could have had more than one pregnancy in this time period. How did the authors handle the data in such cases?
7. I understand the focus of the study is mainly the obstetrical outcomes in the study group. Since arboviral infections like Zika were diagnosed in the study population, can the authors provide data on neonatal outcomes like congenital malformations? For eg, outcomes in zika viral infections have been reported by some of the same authors previously (https://doi.org/10.1136/bmj.k4431)
8. The multivariable analysis for finding out risk factors for adverse perinatal outcomes has been done including variables like gestational diabetes, parity and age. However, considering that the dataset is quite large (around 25,000 women), there could be other clinically intuitive factors like presence of anemia and other co-morbid illnesses like underlying cardiac or renal diseases which could potentially influence the outcomes. Do the authors have data regarding these factors?
9. Discussion section should pertain more to the interpretation of findings from the present study. Much of it seems to have only peripheral relevance to the main findings in the study.

Minor comments

10. What was the definition of sepsis? What infections led to sepsis?
11. Line 236 reads “The median term of fever among those in labor was 37 weeks' amenorrhea”. Idea conveyed is not clear.
12. Ideas conveyed in lines 457-461 are not clear.
13. Lines 105/106 and 108/109 contains same information.
14. Please expand terms like 37WG.
