# Supplementary material for: Causes and consequences of fever in Amazonian pregnant women: A large retrospective study from French Guiana
Source: PLoS Negl Trop Dis. 2023 Oct 24;17(10):e0011721. doi: 10.1371/journal.pntd.0011721 (PMC10624260; doi:10.1371/journal.pntd.0011721)
Supplement: Attachment — Submitted filename: response to reviewer finale 24-09-2023.docx [file pntd.0011721.s003.docx]

Dr HCINI Najeh

Université de Guyane

Service de Gynécologie obstétrique

Centre Hospitalier de l’Ouest Guyanais

97320 Saint Laurent Du Maroni

Tel : 05 94 34 89 39

**Richard A. Bowen
Academic Editor
Andrea Marzi
Section Editor
PLOS Neglected Tropical Diseases**

**PLOS Neglected Tropical Diseases – PNTD-D-23-00920**

Dear Editor, reviewers

We would like to thank the reviewers and Editorial Board for their helpful comments and suggestions regarding our manuscript entitled “**Causes and consequences of fever in Amazonian pregnant women: A large retrospective study from French Guiana.**” by HCINI *et al*. and for providing us with the opportunity to improve it.

Please find below our point-by-point responses to the reviewer comments. Modifications to the original text have been underlined. The reviewer comments are shown in Arial bold. We are also attaching a revised version of the manuscript (with revisions underlined).

We are grateful for your consideration of our revised manuscript.

Sincerely, on behalf of all authors

We are grateful to the reviewers for their critical comments. We have made modifications to the original manuscript and the point-to-point replies and explanations are listed below. We hope that these modifications and our responses adequately address the concerns raised by the reviewers and that the manuscript can be published in this revised version in **PLOS Neglected Tropical Diseases.**

**Reviewer 1:**

**Major comments**

1- ***The authors need to define their study population clearly while analyzing the findings. The objective of study was to study pregnant women with fever. It is mentioned that women were included after 18 weeks of gestation (line 102). However, the results include data from patients in first trimester also (lines 289, 290).***

In our study, we included patients who experienced fever during pregnancy and subsequently delivered after the 18th week. It is important to note that these patients could have encountered fever episodes at any point during the first, second, and/or third trimester. However, it is essential to acknowledge that our retrospective data collection does not provide information on early miscarriages since they are not recorded in the birth register.

We specified in the Methods section:

“Only women who experienced fever and delivered after 18 weeks of gestation were included in the study since the delivery records only encompass deliveries occurring after 18 weeks of gestation (i.e. pregnant women with an early or late miscarriage, before 18 weeks, are not included in the delivery register, and were not included in this study).”

**2- *It is also mentioned in the same lines that fever during labor has been excluded. This was not part of the exclusion criteria mentioned in methods, neither the rationale for this exclusion is explained.***

I appreciate the reviewer's comment and extend my apologies for any potential lack of clarity. Fevers occurring during labor are indeed included in the study population. We have revisited the text in the methods section and made modifications to Figure 1 for improved clarity.

However, in the section describing the identified causes, we provide comprehensive details about the causes of fevers occurring outside labor and delivery. This decision was motivated by the fact that fevers during labor often have different underlying causes and difficult to access in a retrospective chart. To address this point more clearly, we have replaced the term "after exclusion of" with "fever occurring outside labor and delivery" in both the results section and the details of Table 3.

“Table 3: Confirmed Causes of Fever Among Pregnant Women in West French Guiana and Along the Maroni River.

This table only lists cases having occurred outside labor and delivery (n=483).

***3- Did the authors mean fever AFTER delivery? Again, in lines 433-434, we read that intrapartum fever is NOT excluded and there is also a mention of a subgroup analysis, the data on which is missing. Thus, there is no clarity regarding the study population.***

- Again, we apologize for any lack of clarity. To clarify further, intrapartum fever was considered within the scope of the study, whereas fever in the postpartum period was not evaluated as it because it does not directly interfere with the fetal state. Thus, both fever throughout pregnancy and intrapartum fever were considered within the scope of our study as described in figure 1.
- “We also make subgroup analysis” refers to the incidence of fever occurring outside labor and delivery and intrapartum fever. In this study, we have provided the incidence of fever outside labor and intrapartum fever. We have deliberately separated the two groups. The etiologies of fever are very different in each group. These subgroup analyses are now mentioned in the methods: “A descriptive analysis of the causes of fever in the sub-group having fever outside labor and delivery was presented”.
- To clarify, we removed “ In this study, we decided not to exclude these patients to ensure not excluding labor induced by infection and we also make subgroup analysis”

We appreciate the reviewer's feedback and will work to enhance the clarity of the study.

4-***It is not clear whether more than one fever episodes in the same woman has been included or excluded. There were 940 pregnant women with at least 1 episode of fever. It is also mentioned there were 978 infections (line 286).***

We entirely agree with the reviewer that this information may be confusing.

We included women with at least one episode of fever during their pregnancy. Mother with two episode of fever or more were 7.3%. In total there were 978 episodes of fever in 940 mothers.

We have incorporated this clarification into Figure 1:

5. ***Line 297 reads that there were significant differences in pre-existing diabetes between women with and without fever, however this is not substantiated by results provided in Table 2.***

We thank the reviewer for highlighting this error. The text has been revised accordingly. Only gestational diabetes was found to be associated with an elevated risk of fever. Pre-existing diabetes mellitus did not demonstrate any significant association with fever during both pregnancy and the peripartum period within the study population. Therefore, gestational diabetes was analyzed as a potential confounding factor in the multivariate analysis.

***6-How many women were hospitalized?***

Among febrile episode occurring outside labor and delivery (data available in 509), 53% (270/509) were hospitalized with a median stay of 4 days (range: 1 to 33 days). Empiric antibiotics were administered in 38.1% (194/509) of cases.

***7. Table 2 shows the comparison between gestational characteristics of women with and without fever. Although statistically significant differences between gravida and parity is shown between them one wonders how to interpret these findings. Does it mean that younger women with lower parity are predisposed to infections? If so, what are the possible reasons for this? Is it related to differences in the healthcare seeking behaviors in primi versus multigravida women?***

We thank the reviewer for their relevant remarks. We hypothesize that their increased risk can be attributed to their exposure to arboviruses, which were the most prevalent cause of fever in our study, as well as sexually transmitted infections (STIs).

In the case of arboviruses, previous exposure may confer immunity, which could explain the variance in susceptibility among different age and parity groups.

We added in discussion section “We suggest that the increased susceptibility among young parturient individuals may be attributed to their potential exposure to arboviruses, which were the primary cause of fever in our study, in addition to sexually transmitted infections. Regarding arboviral infections (such as DENV, CHIKV, and ZIKV) and chickenpox, prior exposure could potentially confer immunity, contributing to the variance in susceptibility observed.”

***8- The study period is spanning 9 years. It is possible that some women could have had more than one pregnancy in this time period. How did the authors handle the data in such cases?***

We thank the reviewer for their relevant remarks. Yes, it was possible that some patients gave birth multiple times during the 9 years. Indeed, we extensively discussed this issue. However, it became evident to us that various risk factors such as age, parity, obstetric morbidity, and neonatal morbidity varied significantly from one pregnancy to another, and potentially differed between pregnancies with fever and those without. We chose to treat each pregnancy separately. We then considered each delivery as a distinct case. This allowed us to analyze and interpret the results individually for each pregnancy, rather than grouping multiple pregnancies from the same woman together.

We added in methods section “Women who had more than one pregnancy during the study period were counted as separate participants”.

9***-I understand the focus of the study is mainly the obstetrical outcomes in the study group. Since arboviral infections like Zika were diagnosed in the study population, can the authors provide data on neonatal outcomes like congenital malformations? For eg, outcomes in zika viral infections have been reported by some of the same authors previously (***[***https://doi.org/10.1136/bmj.k4431***](https://doi.org/10.1136/bmj.k4431)***)***

We fully agree with the reviewer that this kind of data could enrich our work. The cases of ZIKV infection reported in this research are limited to those with documented fever. However, our decision not to explore the specific effects of each infectious agent in this paper is driven by several considerations.

Firstly, we aimed to avoid redundant information that has been previously discussed, as highlighted in the discussion section. Secondly, providing detailed individual outcomes for each infection (such as syphilis, arboviruses, pyelonephritis, and COVID-19) could potentially make the article overly complex and deviate from our primary objective, which is to offer a broad overview of etiological patterns. Lastly, conducting this type of analysis would necessitate a controlled group with negative cases, which we currently lack. Given all these factors, we have consciously chosen not to incorporate this specific subset of outcomes into our report.

***10- The multivariable analysis for finding out risk factors for adverse perinatal outcomes has been done including variables like gestational diabetes, parity, and age. However, considering that the dataset is quite large (around 25,000 women), there could be other clinically intuitive factors like presence of anemia and other co-morbid illnesses like underlying cardiac or renal diseases which could potentially influence the outcomes. Do the authors have data regarding these factors?***

We appreciate the reviewer's insightful question regarding potential factors influencing adverse perinatal outcomes that were not included in our multivariable analysis.

Several obstetrical factors were tested and reported. As recommended by the reviewer, we included in Table 2 tests for severe anemias (defined as hemoglobin levels below 8 g during pregnancy), renal diseases, and cardiac diseases. Cardiac and renal pathology prior to pregnancy concerns a very small number of patients. We have no data on anemia during pregnancy (defined as hemoglobin below 11 g/dl) in the computerized registry.

We Our findings indicate that there were no statistically significant differences between the two groups for any of the mentioned variables. Additionally, since several factors that could potentially influence the outcomes were present, we conducted a multivariable analysis considering the significant differences identified in the univariate analysis.

However, when we included severe anemias (< 8 g), the results remained unchanged.

11- ***Discussion section should pertain more to the interpretation of findings from the present study. Much of it seems to have only peripheral relevance to the main findings in the study.***

We have revised the discussion section and tried to eliminate concepts that appeared to have limited direct relevance to our results. We hope that the revised discussion will appear more suitable to you. Additionally, we had the article reviewed by a professional editing service.

**Minor comments**

***1-What was the definition of sepsis? What infections led to sepsis?***

Sepsis was defined as life-threatening condition defined as organ dysfunction resulting from infection during pregnancy (World Health Organization Statement on maternal sepsis).

We included this definition in the methods section to enhance clarity.

“Organ dysfunction resulting from infection during pregnancy, as defined by WHO”.

The causes of sepsis are diverse, with urinary tract infections being the predominant factor.

***2-Line 236 reads “The median term of fever among those in labor was 37 weeks' amenorrhea”. Idea conveyed is not clear.***

We modified this sentence to be “The median gestational age at fever onset pre-partum (n=567) was 27 weeks. For women with febrile episodes occurring while in labor (n=411), mean onset was at 37 weeks”. We sincerely appreciate the reviewer's valuable feedback and input.

***3- Ideas conveyed in lines 457-461 are not clear.***

Thank you for pointing this out. The text has been revised.

“Biological diagnosis of infections during pregnancy requires several considerations. For example, for a molecular diagnosis of arboviruses, it is crucial to consider the short window of viral secretion compared to other infections such as cytomegalovirus (34,36). The presence of fever allows the infection onset to be determined with regard to pregnancy term, a critical factor in assessing its impact on the course of pregnancy. Women in their first trimester are particularly at risk (7,36). Therefore, it is necessary to adjust the monitoring protocols to both the underlying pathogen and the timing of infection during pregnancy”.

***4- Lines 105/106 and 108/109 contains same information.***

We thank the reviewer for pointing this out. The text has been revised.

“Only women who experienced fever and delivered after 18 weeks of gestation were included in the study since the delivery records only encompass deliveries occurring after 18 weeks of gestation. [...]. In this study, documented pregnancies with an auricular temperature (infrared emission detection) ≥ 38 °C were included.

5- ***Please expand terms like 37WG.***

We agree with the reviewer and have corrected the text.

**Reviewer #2:**

***The authors have presented an extended retrospective cohort study on the causes of fever in pregnancy in an equatorial setting in South America. The study extends over nine years and thus should represent a realistic picture of the major risk factors of fever in pregnancy for the area. There are a few points for consideration prior to publication.***

We thank the reviewer for their positive comments and are working to improve our manuscript to make it suitable for potential publication.

***Introduction
In the first sentence of the introduction, lines 67 = 68, the authors should include fetal morbidity and mortality as a risk factor of infection during pregnancy.***

We rectified this sentence to be “Severe infections during pregnancy and the post-partum period remain one of the main causes of maternal and fetal morbidity and mortality.”

***Lines 77 – 79: “Although being the origin an endemic area of several epidemics such as arboviruses, there are few information’s about in fever during pregnancy in the Amazon basin” needs to be revised to make English sense.***

Again, we apologize for any lack of clarity.

We write “ While the Amazon region has witnessed various infectious epidemics and continues to face ongoing outbreaks, there is limited data on the causes and consequences of fever in pregnant women in the often-precarious populations of the Amazonian basin”.

Additionally, we had the article reviewed by a professional editing service.
***Line 83: Please change “Scientific data supports” to “Scientific data support” – “data” is the plural form of “datum.”***

Thank you for pointing this out. The text has been revised.
***Methods:
Lines 103 – 114: This whole passage needs to be revised to make sense in English.***

This passage has been revised to read:

“Only women who experienced fever and delivered after 18 weeks of gestation were included in the study since the delivery records only encompass deliveries occurring after 18 weeks of gestation (i.e. pregnant women with an early or late miscarriage, before 18 weeks, are not included in the delivery register, and were not included in this study). In this study, documented pregnancies with an auricular temperature (infrared emission detection) ≥ 38 °C were included.”

***Line 103 – 105: “…documented pregnancies with an auricular temperature taken with an ear thermometer temperature with an infrared emission detection greater than or equal to 38 °C ear temperature with an infrared emission detection thermometer were included” – repetitive information should be excluded.***

Thank you for pointing this out. This has been rectified.

***Line 107 – 109: The exclusion criteria can be included in the flow chart of how subjects were selected for exclusion – see the literature for examples in similar studies.***

Flow chart was modified to include exclusion criteria.
***Line 106: Correct “Monocytogenes” to lower case, and italicise elsewhere in the manuscript as well>***

Thank you for pointing this out. This has been rectified.

***The heading “protocol description” should be changed to diagnosis.***

This modification has been done.

***For the laboratory diagnosis include the manufacturer of the test, city, and country of origin, or if this was developed in-house, reference the methodology/provide primer sequences where relevant.***

This information was added in methods section for GENV, CHIKV, ZIKV, malaria, hepatitis A, SARS-CoV-2, Influenza, syphilis, Coxiella burnetiid, blood culture, leptospirosis.

***What blood culture system was used?***

In the event of a positive blood culture, full identification was performed using either enzymatic or biochemical gallery techniques (such as BIOMERIEUX- VITEK®) or mass spectrometry (such as MALDI Biotyper Sirius by Bruker).

***Define PAMG-1, EBV, CMV and CNGOF– although these are well-recognised acronyms, they are not all exclusive.***

*All acronyms were identified and changed in the text:* Placental alpha 1-microglobulin: (PAMG -1), Epstein-Barr virus (EBV), Cytomegalovirus (CMV), and National College of French Gynecologists and Obstetricians (CNGOF)

***Line 171: Correct the spelling of Listeria monocytogenes and italicise.***

Thank you for pointing this out. This has been rectified.

***Were other bacterial causes of respiratory infection e.g. Haemophilus influenzae other than Hib, tuberculosis, Mycoplasma pneumoniae, or bacterial causes of diarrhoea and dysentery that may be associated with fever, including Salmonella, Shigella or Campylobacter sought, and if so, how?***

Thank you for your valuable feedback. We appreciate your input in helping us improve the clarity of our manuscript. Indeed, we conducted searches to identify bacterial causes of respiratory infections, as well as viral and bacterial causes of diarrhea and dysentery.

For cases of pneumonia, we performed detection tests for Streptococcus pneumoniae and Haemophilus influenzae. The research for tuberculosis was not systematically conducted but guided by the patient's history and clinical findings. In response to your suggestion, we have provided additional details in the Methods section regarding viral and bacterial testing specifically f in case of fever associated with respiratory manifestations.

In cases of febrile diarrhea, we conducted coproparasitological and bacteriological analysis of fecal samples, including testing for Salmonella, Shigella, or Campylobacter, in addition to blood cultures.

We added all this details in the table 1 to clarify methods section.

In the results section, we included specific microorganisms responsible for gastrointestinal infections in Table 2.

***Did blood cultures include examination for typhoid fever, pneumococcal disease, or E. coli bacteraemia (especially secondary to pyelonephritis)? What about leptospirosis, Hepatitis A, malaria, and yellow fever?***

In the event of a positive blood culture, full identification was performed using either enzymatic or biochemical gallery techniques (such as Vitek by Biomérieux) or mass spectrometry (such as MALDI Biotyper Sirius by Bruker). This process included the search for typhoid fever, pneumococcal disease, or E. coli bacteremia, with an antibiogram conducted if the result was positive. The information regarding blood culture was provided in the method section.

All infection details were given in the method section: GENV, CHIKV, ZIKV, malaria, hepatitis A, SARS-CoV-2, Influenza, syphilis, Coxiella burnetiid, blood culture, leptospirosis. Please see revised methods and Table 1.

***Lines 201 – 202: “(living far from hospital, lack or poor pregnancy follow-up, lack of health insurance coverage …) needs to be rephrased to clarify sense.***

Hospitalization was influenced by both clinical presentation and social factors, such as the distance from the hospital, the absence of or insufficient pregnancy monitoring, and the lack of health insurance coverage. Consequently, clinicians may decide to hospitalize patients in cases where they are unable to undergo necessary blood tests due to social constraints, in unattended pregnancies, or for patients coming from foreign countries or living in isolated areas where surveillance is impossible.

***Line 258: “Midwives” or “the midwife”***

Thank you for pointing this out. This has been rectified to be “The midwife”. The midwife routinely entered pregnancy outcomes and complications for all women who had delivered at the West French Guiana hospital into a computerized database.

***Table 1: Correct spelling of Coxiella burnetii and Listeria monocytogenes and italicise.***

Thank you for pointing this out. This has been rectified.

***Did the authors successfully get permission from both the cases and the control group to use the data?***

In this study, we utilized a retrospective descriptive chart review. In accordance with Regulation (EU) 2016/679 of the European Parliament regarding the protection of personal data, a privacy impact assessment for the study was conducted, following the methodology outlined by the French data protection authority (CNIL). This methodology does not require written consent from the patient. Patients who decline the use of their medical data for research purposes were excluded. The study received approval from the hospital's ethics committee.

 Results ***Line 282: What is meant by single deliveries – do the authors mean live births, as per the table some of these were multiple births?***

Thank you for pointing this out. This has been rectified. The test was review.

***Line 283: D the authors mean “same period”?***

Thank you for pointing this out. This has been rectified. We apologize for this error.

***Figure 1: Given the numbers, the study design to select the study participants appears suitable, but may be easier to grasp if the authors could include in the flow chart how many pregnant women presented during the study period in total, and how many were excluded because of refusal to sign consent, insufficient data etc.***

We added in the in our flow chart information in total number of deliveries occurring the study period, patient refusal to use of her medical data, insufficient data.

***Line 295: “Gravidity” may be better than “gravida.”***

Thank you for pointing this out. This has been rectified. We apologize for this error.

***Table 2: Under “Parity” correct “2.8(1-, 4)” – what is the actual range? Correct spelling of “burnetiid” - a burnetiid is an extinct crocodilian reptile and tis error may be a function of the computer’s autocorrect.***

*We apologize for any confusion. We thank the reviewer for his attention. We have corrected this error.*

***Line 356: Do the authors mean “empiric antibiotics?”***

*Yes, we modified “probabilistic antibiotics” to “empiric antibiotics”.*

Discussion and conclusion
***Lines 453 – 454: Fever “was 8.5% in our study population versus 15% in 100 pregnant women of metropolitan France” – can the authors elaborate on this? Is this because they missed cases, had a more stringent definition, or fever is less common in this [population because childhood exposures to common pathogens has increased the population immunity on the area?***

We apologize for the lack of precision in this sentence. Thank you for your valuable feedback. In this context, we are referring to the 'prevalence of unexplained fever.' Our lower rate of fever of unknown origin can be attributed to the comprehensiveness of the examinations we conducted. These examinations encompassed comprehensive laboratory tests, imaging studies, and clinical assessments, which enabled us to identify the causes of fever that might otherwise have remained undiagnosed.

We have carefully re-examined this paragraph and made the necessary changes to ensure that each idea is distinct, and that the general point of view is clearly expressed. “Despite extensive testing, the cause of fever can sometimes remain elusive. The prevalence of unexplained fever may vary depending on the diagnostic procedures conducted in different maternity wards. In our study population, it was found to be 8.5%, while in a sample of 100 pregnant women from metropolitan France, it reached 15%”

***Lines 454 – 462: The authors seem to have conflated a number of ideas in this paragraph, confusing the point they are trying to make. This needs revision.***

Thank you for your valuable feedback. We appreciate your input in helping us improve the clarity of our manuscript. We carefully revisited this paragraph and make the necessary revisions to ensure that each idea is distinct, and the overall point is expressed clearly.

We added: “Despite extensive testing, the cause of fever can remain elusive. The prevalence of unexplained fever may vary depending on diagnostic procedures. In our study population, the cause of fever remained unknown in 8.5% of pregnant women, while in mainland France, this percentage can reach up to 15% (35).

Biological diagnosis of infections during pregnancy requires several considerations. For example, for a molecular diagnosis of arboviruses, it is crucial to consider the short window of viral secretion compared to other infections such as cytomegalovirus.*”*

***The authors have presented a fairly critical view of their study and placed the results in context, regarding the socioeconomic challenges etc. I think it would be important to highlight that their results are very specific to the area, given the increased prevalence of certain regional conditions such as Zika virus. Generally, I this there is scope to expound more on the causes of fever in pregnancy in the area, possibly in a supplementary table if there is no room in the text, as it would then be easier to compare these results with similar studies elsewhere.***

We appreciate the reviewer's valuable feedback. We completely agree that this represents the result in our context. However, despite our thorough literature search using the keyword "fever during pregnancy," no prior studies focusing on fever in pregnant women in the Amazonian region or tropical countries were found. In response to the reviewer's suggestion, we have included Supplementary Table 1, which provides a comparative analysis of the etiologies of fever during pregnancy in Western French Guiana and mainland France.

| Causes of fever | Amazon region  (%) | Mainland France (35)  (%) |
| --- | --- | --- |
| Arboviruses | 30.2 | 0 |
| Urinary tract infections | 27.7 | 21 |
| COVID 19 | 13.2 | - |
| Chickenpox | 5.5 | 0 |
| Influenza | 3.3 | 21 |
| Gastrointestinal infections | 2.9 | 6 |
| Ear, nose, and throat disorders | 2.4 | 2 |
| Pulmonary infections | 2 | - |
| Leptospirosis | 1.8 | 0 |
| Malaria | 1.6 | 0 |
| Sexually transmitted infections | 1.4 | 1 |
| *Coxiella burnetii (Q fever)* | 1 | 0 |
| *Listeria Monocytogenes* | 0.2 | 0 |
| Unknown diagnosis | 8.5 | 15 |
